# Ocular Localization and Transduction by Adenoviral Vectors Are Serotype-Dependent and Can Be Modified by Inclusion of RGD Fiber Modifications

**DOI:** 10.1371/journal.pone.0108071

**Published:** 2014-09-18

**Authors:** Kazuhiro Ueyama, Keisuke Mori, Takuhei Shoji, Hidekazu Omata, Peter L. Gehlbach, Douglas E. Brough, Lisa L. Wei, Shin Yoneya

**Affiliations:** 1 Department of Ophthalmology, Saitama Medical University, Moroyama, Iruma, Saitama, Japan; 2 Wilmer Eye Institute, Johns Hopkins University School of Medicine, Baltimore, Maryland, United States of America; 3 GenVec, Inc., Gaithersburg, Maryland, United States of America; 4 National Eye Institute, Bethesda, Maryland, United States of America; Swedish Medical Center, United States of America

## Abstract

**Purpose:**

To evaluate localization and transgene expression from adenoviral vector of serotypes 5, 35, and 28, ± an RGD motif in the fiber following intravitreal or subretinal administration.

**Methods:**

Ocular transduction by adenoviral vector serotypes ± RGD was studied in the eyes of mice receiving an intravitreous or subretinal injection. Each serotype expressed a CMV-GFP expression cassette and histological sections of eyes were examined. Transgene expression levels were examined using luciferase (Luc) regulated by the CMV promoter.

**Results:**

GFP localization studies revealed that serotypes 5 and 28 given intravitreously transduced corneal endothelial, trabecular, and iris cells. Intravitreous delivery of the unmodified Ad35 serotype transduced only trabecular meshwork cells, but, the modification of the RGD motif into the fiber of the Ad35 viral vector base expanded transduction to corneal endothelial and iris cells. Incorporation of the RGD motif into the fiber knob with deletion of RGD from the penton base did not affect the transduction ability of the Ad5 vector base. Subretinal studies showed that RGD in the Ad5 knob shifted transduction from RPE cells to photoreceptor cells. Using a CMV-Luc expression cassette, intravitreous delivery of all the tested vectors, such as Ad5-, Ad35- and Ad28- resulted in an initial rapid induction of luciferase activity that thereafter declined. Subretinal administration of vectors showed a marked difference in transgene activity. Ad35-Luc gene expression peaked at 7 days and remained elevated for 6 months. Ad28-Luc expression was high after 1 day and remained sustained for one month.

**Conclusions:**

Different adenoviral vector serotypes ± modifications transduce different cells within the eye. Transgene expression can be brief or extended and is serotype and delivery route dependent. Thus, adenoviral vectors provide a versatile platform for the delivery of therapeutic agents for ocular diseases.

## Introduction

There are several vector platforms currently under investigation for ocular gene therapy. [1]–[3] Among them, adenoviral vectors have several attractive properties, including high transduction efficiency, transduction of a wide spectrum of dividing and non-dividing cells and absence of random integration into the host genome. Two phase I clinical trials using adenoviral vector have previously been conducted in 2005 and 2006, one in a pediatric population, and the other in an elderly population. [4], [5] These two trials showed that adenoviral vector-mediated ocular gene transfer is safe and a viable approach for the treatment of ocular diseases. However, since adenoviral vector serotype 5 (Ad5) is highly prevalent in the human population, many individuals have been exposed to Ad5 and subsequently, have generated a dose-dependent neutralizing antibody response. This antibody response toAd5 is thought by many to contribute to a shorter duration of transgene expression, which is considered to be a disadvantage of adenoviral vector therapy. [6]–[8] In devising strategies to circumvent this vector limitation, vector design modification and/or alternative serotype usage may be a means to improve the utility of adenoviral vectors as a drug delivery system. Especially, Ad5 has a limitation in transgene expression so modification was required [9].

There are several subgroups of adenovirus serotypes which have differing receptors. Ad5 in subgroup C binds to the coxsackie virus adenovirus receptor (CAR), while adenoviral vector serotype 35 (Ad35) in subgroup B, binds to CD46 (also known as membrane co-factor protein, MCP). [10]–[12] CD46 is a member of the soluble membrane complement regulatory protein. CD46 is expressed on most nucleated human cells and acts to protect the host cell against autologous complement attack by degrading C3b. [13], [14] Yang and associates reported CD46 up-regulation by inflammatory cytokines, including TNF-α and IL-1ß, and with stimulation of hydroquinone, an important oxidant in cigarette smoking. [15] Johnson and associates showed that CD46 localizes in some drusen and RPE cells overlying drusen. [16] These findings suggest that CD46 is a molecular target contributing to the pathogenesis of age-related macular degeneration.

Incorporation of the sequence arginine-glycine-aspartic acid (cyclic RGD) into the knob portion of the adenovirus vector promotes binding to the cell surface and is mediated by integrin αVß3/5 receptors. [17] Therefore Ad35 with RGD in the fiber knob retains its native tropism to CD46 and also has enhanced binding to cell surface integrin αVß3/5 receptors. Since neutralizing antibodies are reduced in response to Ad35 as compared to Ad5, [18] we hypothesized that host immune response to Ad35 is reduced and that this could result in prolonged gene expression. Based on this hypothesis, a group of us, Hamilton, et. al., investigated the duration of expression and efficacy of Ad35 and reported that pigment epithelium-derived factor (PEDF) gene expression delivered by intravitreous injection of Ad35, prolonged PEDF expression as compared to the same gene delivered by Ad5. [19] In the described studies herein, we strategically designed and produced adenoviral vectors of differing serotypes, including Ad5, Ad35 and adenoviral vector serotype 28 (Ad28), with or without modifications within the fiber knob and/or penton base, which is also important for vector binding and internalization. Vector transduction efficiency, latency and intraocular tropism were then directly compared in order to identify vector modifications that may improve transgene expression kinetics and provide the basis for the development of therapeutic protein agents that are applicable to the pathogenesis and treatment of ocular diseases such as age-related macular degeneration.

## Methods

### Animals

Two hundred and eighteen female C57BL/6 mice were acclimated for approximately one week prior to use at 6–8 weeks of age. The animals were housed under controlled lighting conditions (12 hrs:12 hrs light/dark), and were given food and water *ad libitum* throughout the experiments. The animals were anesthetized by intramuscular injection of 80 mg/kg of ketamine hydrochloride. The pupils were dilated with 0.5% tropicamide and 0.5% phenylephrine hydrochloride. All animals were treated under deep sedation and euthanasia. The procedures were performed in accordance with the Association for Research in Vision and Ophthalmology resolution on the use of animals in research. This study was also approved by the Institutional Review Board of Animal Care of Saitama Medical University.

### Production of Adenoviral Vectors of Serotypes 5, 35 and 28 Expressing Green Fluorescent Protein or Luciferase

**Figure 1:**
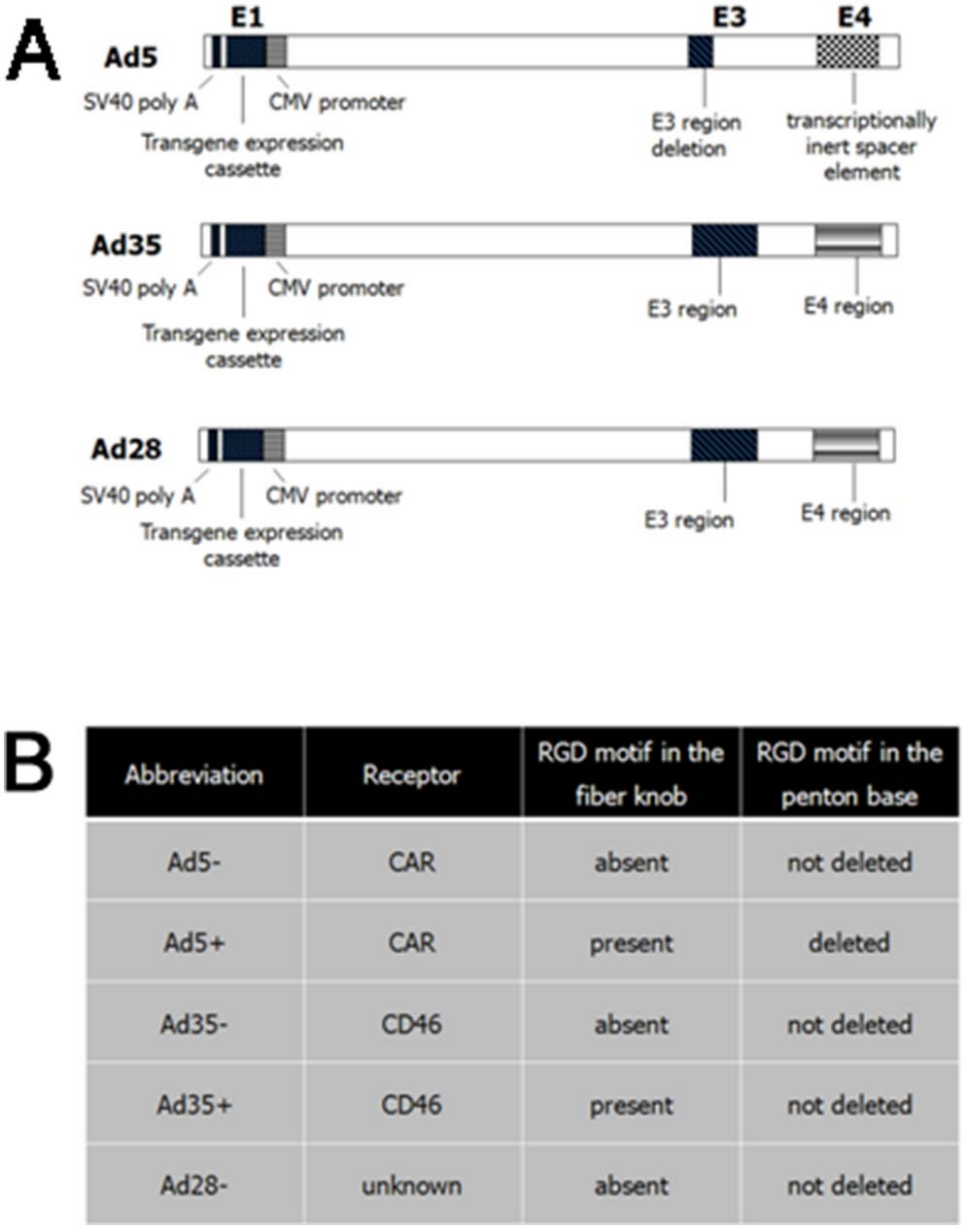
Schematic diagram and abbreviation of Ad5, Ad35 and Ad28. Schematic diagram comparing similar regions in Ad5, Ad35 and Ad28 vectors (**A**). The E1 region has been deleted in each construct and replaced with the SV40 poly A region, the luciferase or green fluorescent protein cDNA, and the cytomegalovirus promoter (CMV). In the Ad5 vector, there is a partial E3 deletion and the E4 region was replaced with a transcriptionally inert sequence. In the Ad35 and Ad28 vectors, the E3 regions and E4 regions are intact. The genomes of all vectors are approximately the same size. *Plus* in the vector abbreviation means RGD motif incorporation in the fiber knob. Ad5+ alone has also been deleted for the RGD motif in the penton base (**B**).

Production and quantification of Ad5, Ad35 and Ad28 has been described in detail elsewhere. [20]–[24] All adenoviral vectors were constructed using GenVec’s AdFAST technology, which employs homologous recombination methods in *Escherichia coli* to quickly restructure the adenoviral vector genome. The E1 region has been deleted in each construct and replaced with the SV40 poly A region, the luciferase (Luc) or green fluorescent protein (GFP) cDNA, and the cytomegalovirus promoter (CMV). The expression cassettes are oriented such that the transgenes are transcribed from right to left relative to the adenovirus genome. Vectors without transgene cDNA were also prepared for each serotype as a Null vector control. During construction, an RGD-4C peptide was incorporated into the HI loop of the vector fiber knob, as described previously. [25]–[27] There is a partial E3 deletion, and the E4 region of Ad5 was replaced with a transcriptionally inert sequence. The E3 regions and E4 regions of Ad35 and Ad28 are intact. The genome of all vectors is approximately the same size (**Figure 1A**). Each adenoviral vector and its abbreviation is summarized in **Figure 1B**. ‘*Plus*’ in the vector abbreviation means with the RGD motif incorporated in the fiber knob and ‘*minus*’ means without incorporation. Ad5+ alone has also been deleted for the RGD motif in the penton base. The ‘plus’ vector is limited to Ad5 and Ad35 expressing GFP cDNA. The following are the thirteen vectors used in this experiment, Ad5+GFP, Ad5-GFP, Ad35+GFP, Ad35-GFP, Ad28-GFP, Ad5-Luc, Ad35-Luc, Ad28-Luc, Ad5+Null, Ad5-Null, Ad35+Null, Ad35-Null and Ad28-Null. The receptors for Ad5 and Ad35 are known as CAR and CD46, respectively. [10]–[12] The receptor for Ad28 remains an active area of research and has yet to be identified.

### Intraocular Injection Procedures of Adenoviral Vectors

Adult C57BL/6 mice were given either an intravitreous injection of 10^9^ particles (pu) of adenoviral vectors or a subretinal injection of 10^8^ particles of adenoviral vectors in each eye. Mice were anesthetized, the pupils were dilated, and under a dissecting microscope, an injection was performed with a microinjector (IM-6; Narishige Scientific Instrument Lab., Tokyo, Japan). The drawn capillary glass needle was passed through the sclera at the equator into the vitreous cavity. Each intravitreous injection occurred with direct observation of the glass capillary tip located in the center of the vitreous cavity. A subretinal injection was performed using a condensing lens system on the dissecting microscope that allowed visualization of the retina during the injection. The pipette tip was passed through the sclera and was positioned beneath the surface of the retina. Following injection, a subretinal bleb was created that was uniform in size. A rare eye with intraocular hemorrhage, lens trauma, or other complication occurring during the vector injection procedure was excluded, at the time of injection, from this study.

### Visualization of GFP Expression by Fluorescent Microscope

To visualize the intraocular GFP expression after intravitreous and subretinal injections of AdGFP, eyes were enucleated under deep anesthesia one day after the injection. All eyes were immediately fixed in 4% paraformaldehyde in phosphate buffered saline (PBS) for 60 minutes. After rinsing with PBS the eyes were oriented in optimum cutting temperature embedding compound (OCT; Sakura Finetek, Torrance, USA) with the cornea facing forward and with 12 o’clock positioned superiorly and then snap frozen in liquid nitrogen after which they were stored at –80 C until sectioning. Frozen sections (10 µm) of eyes were counter-stained by propidium iodide (PI, Sigma-Aldrich, St. Louis, MO, USA). The sections were observed using the fluorescent microscope (Axio Imager 2, Carl Zeiss, Oberkochen, Germany). 3 eyes each of Ad5+, Ad5-, Ad35+, Ad35-, Ad28- intravitreous and subretinal injected eyes (30 eyes) and 3 eyes of control (total 33 eyes) were enucleated at this study to visualize the intraocular GFP expression.

### Analysis or Measurement of Luciferase Activity

Injected eyes were enucleated on Day 1, 7, 14, 28 and 180 and luciferase activity measured. Frozen whole eye specimens were ground and analyzed with a luciferase assay system (Promega Systems, Madison, WI). Ocular homogenates were lysed with 300 µL 1x Reporter Lysis Buffer (Promega, Madison, WI). Resultant lysates were analyzed with a luciferase assay system according to the manufacturer’s protocol (Promega). The total ocular protein concentration was determined by Bradford dye binding procedure with a protein assay (Bio-Rad, Hercules, CA) to normalize the measurement of luciferase activity. 147 eyes (72 intravitreous injection and 75 subretinal injection) were analyzed luciferase activity at this study.

## Results

### Ocular Localization by Various Adenoviral Vector Serotypes

**Figure 2:**
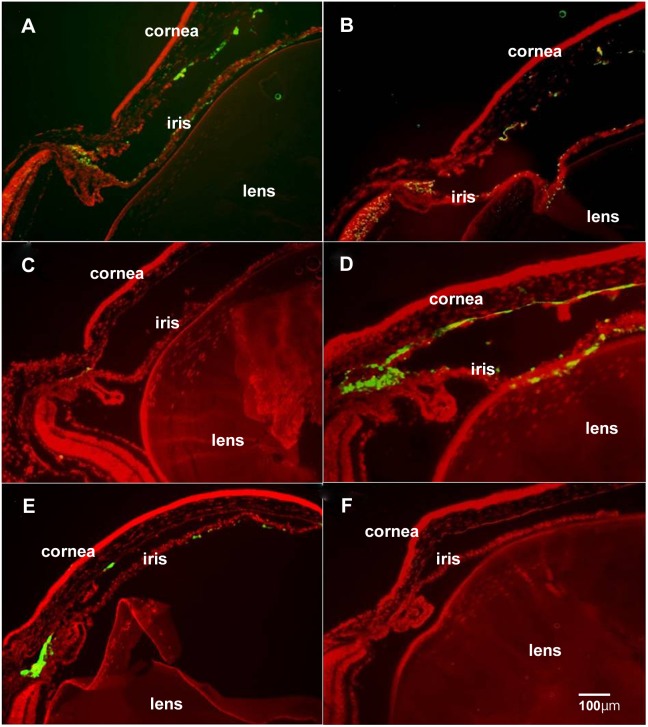
Intraocular localization of GFP delivered by intravitreous injections. Ad5-GFP vectors transduced corneal endothelial cells, trabecular cells and iris cells (**A**). Ad5+GFP vectors transduced corneal endothelial cells, trabecular cells and iris cells (**B**). Ad35-GFP vectors transduced travecular cells alone (**C**). Ad35+GFP vectors transduced corneal endothelial cells, trabecular cells and iris cells (**D**). Ad28-GFP vectors transduced corneal endothelial cells, trabecular cells and iris cells (**E**). An Ad28-Null injected eye showed no GFP fluorescence in any ocular cells (**F**). Scale bar = 100 µm.

To assess whether different adenoviral vector serotypes transduced the same or different ocular cells, we undertook a series of studies using Ad5, Ad35, and Ad28 vectors containing the Green Fluorescent Protein (GFP) transgene. Adult female C57BL/6 mice were injected either intravitreally or subretinally. In intravitreal delivery studies, Ad5 and Ad5-GFP vectors transduced corneal endothelial cells, trabecular and iris cells (**Figure 2A**). Ad5+GFP vectors transduced corneal endothelial cells, trabecular and iris cells (**Figure 2B**). Ad35-GFP vectors only transduced trabecular cells (**Figure 2C**). Ad35+GFP vectors transduced corneal endothelial cells, trabecular and iris cells (**Figure 2D**). Ad28 vectors transduced corneal endothelial cells, trabecular and iris cells (**Figure 2E**). The negative control, all AdNull vectors without transgene cDNA, showed no fluorescence in any ocular cells (**Figure 2F**). Incorporation of the RGD motif into the fiber knob with deletion of RGD from the penton base did not affect the transduction ability of the Ad5 vector base (**Figure 2A, 2B**). However, the modification of the RGD motif into the fiber of the Ad35 viral vector base expanded transduction to corneal endothelial and iris cells (**Figure 2C, 2D**).

**Figure 3:**
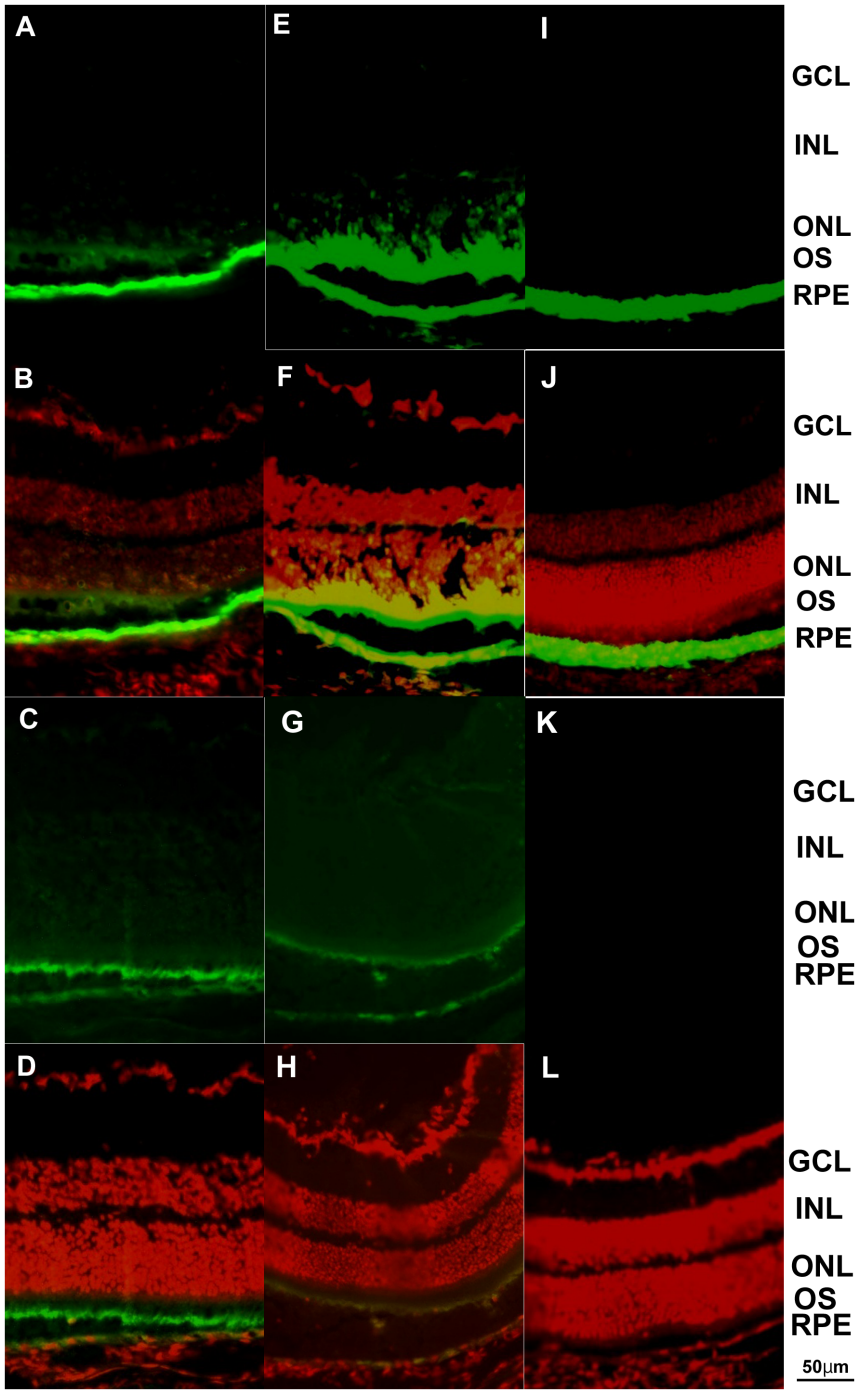
Intraocular localization of GFP delivered by subretinal injections. Ad5-GFP injected eyes showed GFP fluorescence in the photoreceptors and RPE cells (A, B). Ad5+GFP injected eyes also showed GFP fluorescence in the photoreceptors and RPE cells (C, D). Ad35-GFP injection also showed GFP fluorescence in the photoreceptors and RPE cells (E, F). Ad35+GFP injection also showed GFP fluorescence in the photoreceptors and RPE cells (G, H). Ad28- transduced RPE cells alone (I, J). Ad28-Null injected eye demonstrated no obvious fluorescence (K, L). Ad5-GFP injected eyes demonstrated stronger GFP fluorescence in the RPE cells (A, B), and Ad5+GFP transduced photoreceptors dominantly (C, D). Scale bar = 50 µm.

Subretinal injection of Ad5- vectors with GFP showed green fluorescence in photoreceptors and RPE cells (**Figure 3A, 3B**). Also, subretinal injection of Ad5+ vectors with GFP showed green fluorescence in photoreceptors and RPE cells (**Figure 3C, 3D**). Ad35-GFP vectors also transduced photoreceptors and RPE cells (**Figure 3E, 3F**). Ad35+GFP vectors also transduced photoreceptors and RPE cells (**Figure 3G, 3H**). Eyes injected subretinally with Ad28-GFP resulted in transduction of RPE cells alone (**Figure 3I, 3J**). GFP fluorescence was not observed in AdNull injected eyes (**Figure 3K, 3L**), thus indicating that the observed green fluorescence was not due to nonspecific fluorescence. The addition of the RGD motif in the fiber and deletion in the penton base resulted in the same cells transduced but the proportion changed, with a greater intensity of GFP in the photoreceptors than RPE cells (**Figure 3A, 3B, 3C, 3D**). However, in the case of Ad35 vectors with GFP, the RGD motif did not modify the ocular specificity of GFP expression. Both Ad35-GFP and Ad35+GFP vectors transduced photoreceptors and RPE cells (**Figure 3E, 3F, 3G, 3H**).

### Pharmacokinetic Analysis of Transgene Expression by Alternate Adenovector Serotypes

**Figure 4:**
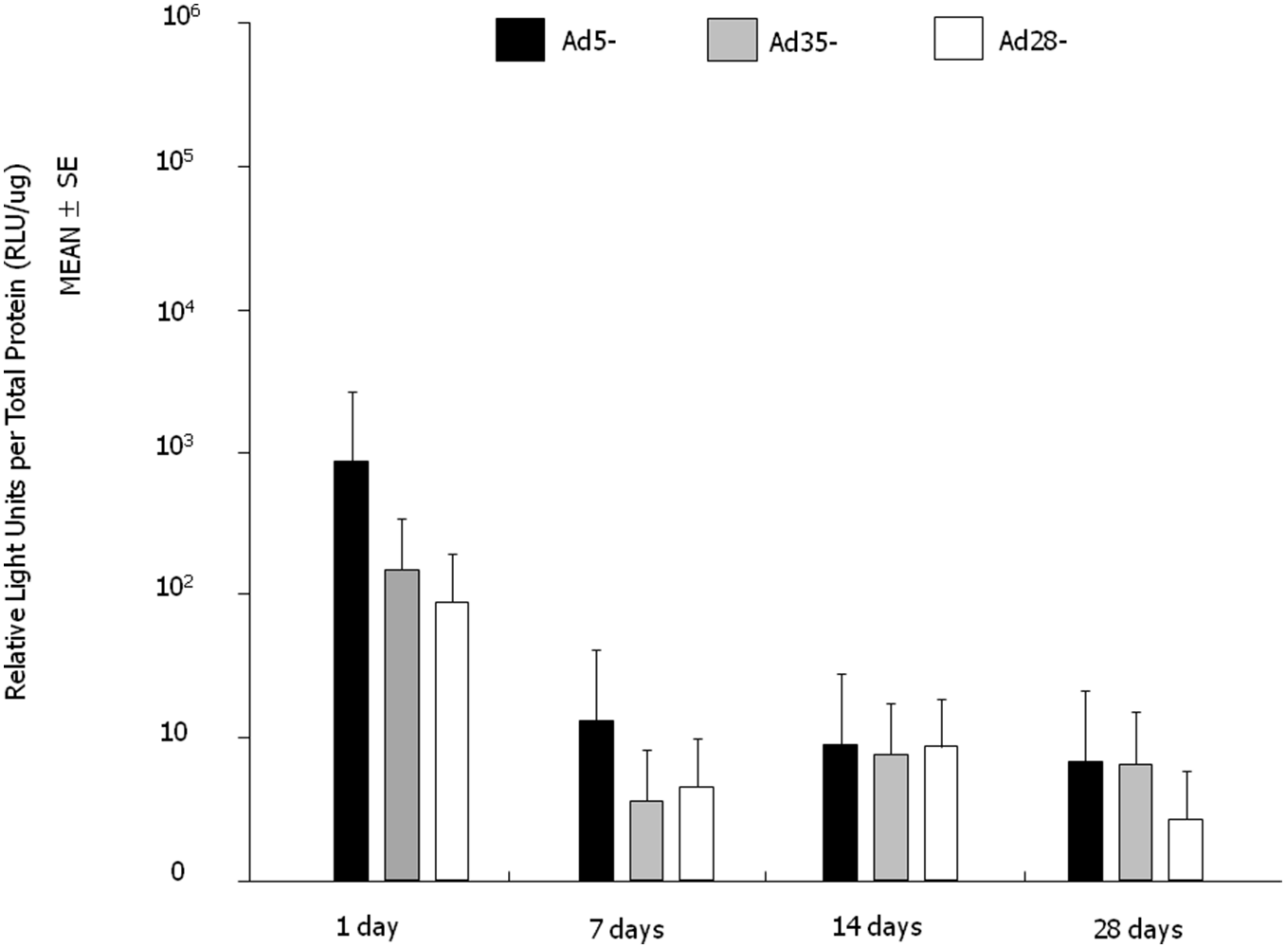
Luciferase activity following single intravitreous injections of Ad5-Luc, Ad35-Luc and Ad28-Luc vectors. Luciferase activity peaked for all vectors within one day post-injection. Of all the tested vectors, the Ad5-Luc vector gave the greatest luciferase activity at Day 1. After Day 1, luciferase activity declined for all constructs and by Day 28 relative luciferase activity was very similar between the three vectors. Error bars: SEM. n = 6/vector/time point.

**Figure 5:**
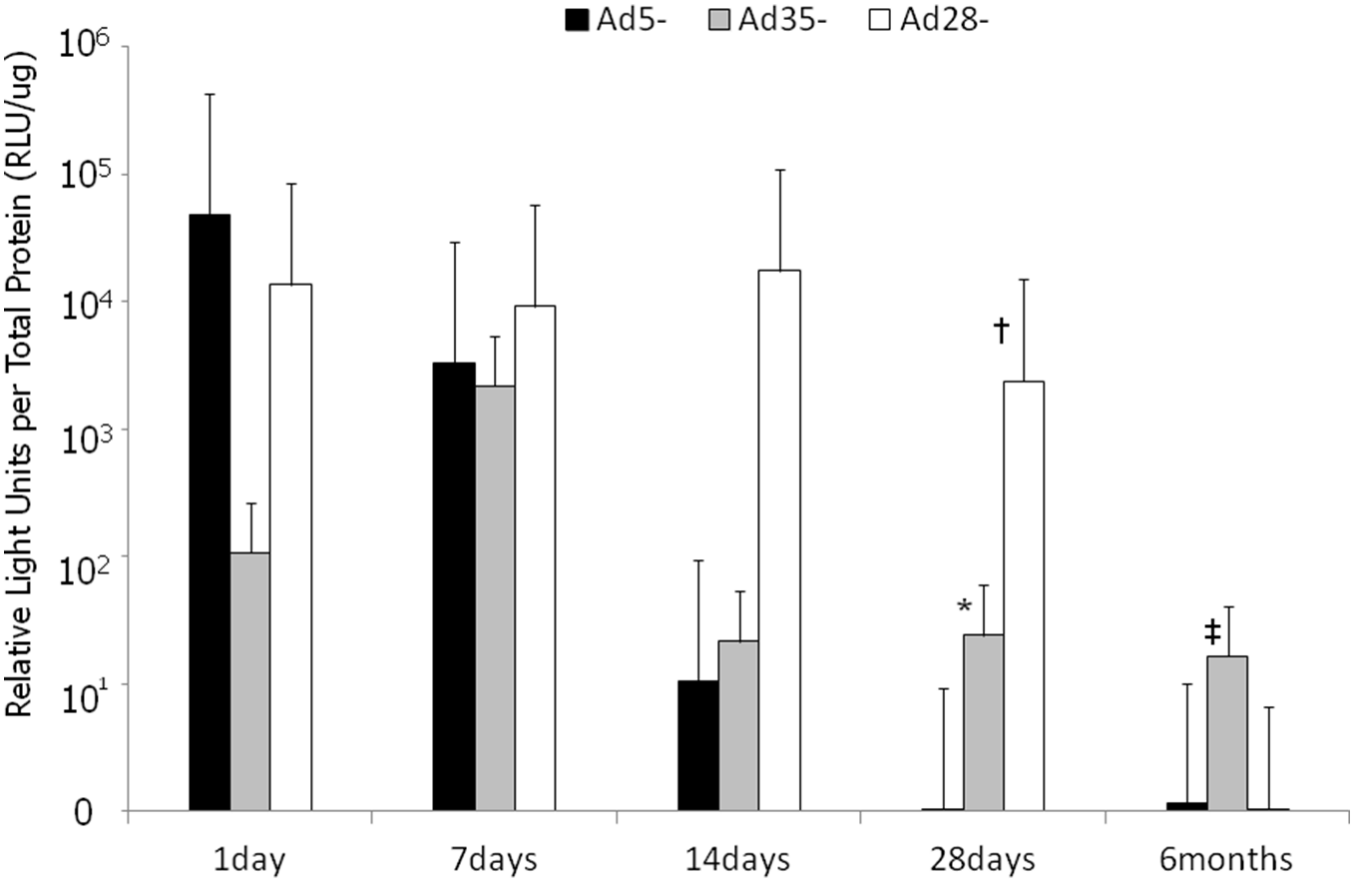
Luciferase activity following a single subretinal injection of Ad5-Luc, Ad35-Luc and Ad28-Luc. Ad5-Luc expression peaked one day after injection, but thereafter decreased quickly. Ad35-Luc expression peaked at seven days after injection and remained elevated out to 6 months. Ad35-Luc expression was significantly stronger than Ad5-Luc at the time point of 28 days and 6 months (*p* = 0.0127*, 0.033^‡^, respectively). Ad28-Luc expression was substantial and was sustained throughout one month of observation, but was low at 6 months. Ad28-Luc expression was significantly stronger than Ad5-Luc at 28 days after injection (*p* = 0.0127^†^). Error bars: SEM. n = 4 to 6/vector/time point. Which are Ad5-1day 4eyes, Ad35-1day 5 eyes, Ad28-1day 4 eyes, Ad5-7days 5 eyes, Ad35-7days 4 eyes, Ad28-7days 5 eyes, Ad5-14days 4 eyes, Ad35-14days 5 eyes, Ad28-14days 4 eyes, Ad5-28days 5 eyes, Ad35-28days 4 eyes, Ad28-28days 4 eyes, Ad5-6months 6 eyes, Ad35-6months 4 eyes, Ad28-6months 6 eyes.

We next evaluated the transgene expression profile from Ad5, Ad35, and Ad28 adenoviral vectors using the CMV promoter and the marker transgene, luciferase. At intravitreous administration, 6 eyes were measured at each serotypes and each time point (72 eyes). Following a single intravitreous administration of 10^9^ particles per eye, the Ad5 vector gave the greatest induction in luciferase activity within 24 hours post-administration. By day 28, all three serotypes, Ad5, Ad35 and Ad28 gave similar levels of luciferase activity (**Figure 4**). Interestingly, a 10-fold lower dose of adenoviral vectors delivered subretinally, resulted in luciferase levels 10–1000 fold higher than eyes injected intravitreously with 10^9^ particles per eye. At subretinal administration, 4 to 6 eyes were measured at each serotypes and each time point (75 eyes). Subretinal administration of Ad5-Luc administration resulted in a rapid increase in luciferase activity at 24 hours declining by day 14. Ad35-Luc activity peaked at day 7 and thereafter declined, but by day 28 levels (n = 4, mean 24.2) were greater than that of Ad5-Luc (n = 5, mean 0.084) (Mann-Whitney U test, *p* = 0.0127). Ad28-Luc vectors also resulted in a rapid increase in marker gene activity in the first 24 hours and remained high till day 28. Ad28-Luc (n = 4, mean 2390) expression was significantly stronger than Ad5-Luc (n = 5, mean 0.084) on 28 days after injection (Mann-Whitney U test, *p* = 0.0127). Ad35 vectors though gave the most prolonged gene expression profile with elevated levels even after 6 months. Ad35-Luc (n = 4, mean 16700) expression was significantly stronger than Ad5-Luc (n = 6, mean 416) at the time point of 6 months (Mann-Whitney U test, *p* = 0.033) (**Figure 5**). Summary of the localization and duration of transgene expression of intravitreous injection of Ad5-, Ad35-, Ad28-, and subretinal injection of Ad5-, Ad35-, Ad28- are shown in **Table 1**. These data show that as a class of molecules, adenoviral vectors, transduce many ocular cell types and that depending upon the specific serotype and administration route can give different pharmacokinetic profiles, thus, further emphasizing the utility and versatility of adenoviral vectors as a delivery system for therapeutic products for ocular diseases.

**Table 1:** Summary of the localization and duration of transgene expression.

Route of administration	Abbreviation	Localization	Duration of transgene expression
Intravitreous injection	Ad5-	Corneal endothelial cells,Trabecular cells, Iris cells	Peaked within one day post injection
Intravitreous injection	Ad35-	Trabecular cells	Peaked within one day post injection
Intravitreous injection	Ad28-	Corneal endothelial cells,Trabecular cells, Iris cells	Peaked within one day post injection
Subretinal injection	Ad5-	Photoreceptors, RPE cells(stronger GFP fluorescencein the RPE cells)	Peaked one day after injection, but decreased quickly
Subretinal injection	Ad35-	Photoreceptors, RPE cells	Peaked at seven days after injection and remained elevated out to 6 months
Subretinal injection	Ad28-	RPE cells	Substantial and sustained throughout one month, but low at 6 months

## Discussion

In this study we evaluated transgene expression and intraocular localization of transgene products following delivery of adenoviral vector serotypes 5, 35 and 28. There are several endocytic routes by which viruses are internalized into cells: clathrin-mediated endocytosis, caveolar endocytosis, and clathrin-and caveolae-independent endocytosis. Some viruses are internalized into cells by clathrin- and caveolae-independent pathways such as dynamin-independent, microfilament-independent, clathrin- independent, lipid raft-dependent, and macropinocytotic pathways. Dynamin is a large GTPase that plays a key role in “pinching off” coated vesicles to form coated pits during classical clathrin-mediated endocytosis. [28] These various internalization pathways may influence the fate of different Ad serotypes administered by different routes.

Eyes with intravitreous injection of Ad5-GFP, Ad5+GFP, Ad28-GFP, and Ad35+GFP demonstrated GFP fluorescence in the iris, corneal endothelial and trabecular cells, but little in the retinal cells. It is previous reported that recombinant adenovirus transduction to the cornea was seen in epithelial layers and endothelial cells. [29] Eyes with subretinal injection of Ad5-GFP demonstrated strong GFP fluorescence in the RPE cells that is consistent with previous reports. [30]–[33] In eyes injected subretinally with Ad5+GFP vectors, photoreceptor cells showed greater GFP fluorescence than RPE cells. Both Ad35+GFP and Ad35-GFP injected eyes showed comparable fluorescence in both photoreceptors and RPE cells. Ad28-GFP showed expression predominantly in the RPE cells. The vector tropism evident following subretinal delivery may be influenced by intraretinal localization of receptors for each serotype as well as fiber modifications. Collectively, these studies demonstrate that adenoviral vectors transduce a wide variety of ocular cell types though each serotype specifically transduces selective cell types. The inclusion of an RGD in the fiber in some cases can broaden the transduction properties. Thus, the availability of a wide array of adenoviral vector serotypes provides a cadre of delivery vehicles that can be “directed” to specific ocular cell types. These experiments demonstrate that fiber modifications, such as RGD or other, can be further utilized to enlarge the number of ocular cell types transduced or to preferentially target one cell type over another. These studies demonstrate the flexibility and feasibility of engineering adenoviral vectors for ocular disease applications.

An obvious difference observed between Ad5-GFP and Ad5+GFP is photoreceptor tropism. Cashman et al. demonstrated that deletion of the RGD domain in the penton base coupled with use of the CBA promoter permitted transgene expression in neural retina approximately 667 times more efficiently than the conventional nonmodified Ad5. [34] Sweigard et al. also reported that the combination of opsin promoter and the deleted RGD motif in the penton base resulted in stronger transduction to the photoreceptor. [35] The results of the present study with Ad5+GFP and Ad5-GFP are consistent with those reports. Our Ad5+GFP construct is designed to incorporate the RGD motif in the fiber knob, but delete for the RGD motif in the penton base. Taken collectively, deletion of the RGD motif in the penton base may be a key factor leading to photoreceptor cell transduction. Conversely, several lines of evidence indicate that interaction of αV integrin with the penton base promotes efficient uptake of virus into cell endosomes. [36] Mallam et al. reported that the expressions of CAR and αV integrin are evident in the RPE and throughout the retina exclusive of the photoreceptor outer segments. [37] They described the reduced transduction efficiency with conventional Ad5 (corresponding to Ad5-GFP in this experiment) in photoreceptors and suggested steric hindrance caused by the tight packing of photoreceptor outer segment as potential mechanism of interference with effective binding of the large fiber of conventional Ad5 to inner segment CAR. One possible explanation for the paradoxical tropism of Ad5+GFP to photoreceptors is that the RGD motif deletion in the penton base may result in reduced viral uptake in RPE cells, following diversion of the vector to the contiguous photoreceptors. However, weak but distinct tropism of Ad5+GFP to RPE cells (**Figure 3C, D**) suggests an as yet unknown mechanism for viral binding and insertion into photoreceptors and RPE cells.

In contrast to CAR and αV integrin, CD46 localizes to both RPE cells and the photoreceptor inner and outer segments. [37] The characteristics of CD46 distribution could potentially mediate Ad35 transduction to both the photoreceptors and the RPE cells following subretinal delivery. In this work there was no obvious additional effect on tropism of Ad35 vectors with RGD motif incorporated into the fiber knob. This may be because the native tropism of Ad35 to its CD46 receptor is stronger than the influence imparted by fiber modification in this experimental setting, using normal murine eyes.

CD46 acts as a serum protease cofactor that degrades C3b and prevents activation of the complement cascade that serves to protect the host cell against autologous attack. [38] CD46 is present in drusen associated RPE cells, as well as in small, spherical substructural elements within drusen. [16] Increased membrane complement regulatory proteins such as CD46, CD55, and CD59 may help to protect RPE cells from complement and oxidant mediated injury in diseases such as age-related macular degeneration. [15] These facts suggest an interesting hypothesis that Ad35 vectors may accumulate in drusen and related subretinal pathologies mediated by CD46. Should this hypothesis be supported it could provide evidence for pathology targeted delivery of transgene constructs.

It is well known that integrin aVß3 is required for angiogenesis and that αVß3 expression is increased during angiogensis. [39], [40] Therefore, enhanced vector transduction, mediated by integrin αVß3, may be possible in neovascular tissue. Using the murine ischemic retinopathy model, simulating retinopathy of prematurity, we have previously demonstrated selective targeted transduction of retinal neovascular tissue following injection of conventional Ad5 vector. [33] Although there was no significant difference in tropism between Ad35+GFP and Ad35-GFP delivered subretinally in normal murine eyes, these findings may support the premise that Ad35 with RGD motif incorporation into the fiber knob promotes vector binding to molecules involved in angiogenesis and complement activation. Further study is warranted to investigate Ad35 vector transduction efficiency and localization in animal models of intraocular neovascularization, drusen and age-related macular degeneration-related pathologies.

Although Ad concentration delivered via intravitreous injection was 10-fold higher than that of subretinal injection, whole eye luciferase expression was 10 to 1000-fold higher in eyes with subretinal injection for all vectors examined. With intravitreous injection, luciferase expression with Ad5-Luc was strongest within one day following injection, but then declined to similar levels as that of Ad35-Luc injected eyes. With subretinal injection, Ad5-Luc expression peaked within one day after injection, but decreased quickly. Ad35-Luc expression peaked at 7 days after injection but, was prolonged to 6 months. Ad28-Luc expression was sufficient and sustained through one month but was decreased after that.

As replicated in this study a primary limitation of the Ad5 vector is that it does not provide long-term expression. The present study supports this finding using Ad5-GFP constructs. Meanwhile, Ad35 showed greater levels of expression and a more sustained expression, out to six months in this work, following subretinal injection. Mallam et al. also found 8-month GFP expression with subretinal delivery of Ad5 with an Ad35 fiber knob.^37^ Taken together, either the Ad35 vector or adenoviral vector with an Ad35 knob may each provide an advantage over the conventional Ad5 vector with the respect to duration of transgene expression.

In conclusion, our data indicate that adenovirus serotype and fiber modification contribute to vector transduction and duration of expression in murine retinal cells. Transduction efficiency over time has also differed by serotype especially when delivered subretinally. These data are relevant for the use of adenovirus-based gene transfer vectors to target specific cells, and also for achieving long-term gene expression in the eye. The potential for significant transgene expression for time periods exceeding six months but not life-long, could be beneficial in the management of ocular neovascular diseases.
